# Standalone anteroposterior contrast spread pattern is insufficient to distinguish lumbar epidural from extradural spread: A prospective study

**DOI:** 10.1016/j.inpm.2025.100634

**Published:** 2025-08-25

**Authors:** Afrin Sagir, Thomas T. Simopoulos, Jyotsna V. Nagda, Alexandra C.G. Fonseca, Viet L. Cai, Nasir Hussain, Chen Liang, Jatinder S. Gill

**Affiliations:** a Department of Anesthesiology and Critical Care, Hospital of the University of Pennsylvania, Penn Center for Perioperative Outcomes Research and Transformation, University of Pennsylvania, Perelman School of Medicine, 3400 Spruce Street, Suite 680 Dulles, Philadelphia, PA, 19104, USA; b Department of Anesthesiology, Critical Care and Pain Medicine, Beth Israel Deaconess Medical Center, Harvard Medical School, 330 Brookline Ave, Boston, MA, 02215, USA; c Department of Anesthesiology, Perioperative and Pain Medicine, Brigham and Women's Hospital, Harvard Medical School, 75 Francis Street, Boston MA 02115, USA; d Department of Anesthesiology, The Ohio State University, Wexner Medical Center, 410 W 10th Ave, Columbus, OH 43210, USA; e NYU Langone Health, 550 First Avenue, New York, NY 10016, USA

## Abstract

**Background:**

There is debate about whether a standalone anteroposterior (AP) view can distinguish epidural contrast from non-epidural contrast spread.

**Objectives:**

This study aims to assess the accuracy of the AP (anteroposterior) and Contralateral Oblique (CLO) views in distinguishing epidural contrast spread patterns from non-epidural contrast spread patterns.

**Methods:**

Patients undergoing lumbar epidural steroid injections consented to participate in the study. A 20-gauge Tuohy needle was advanced very close to the epidural space, and 0.5–1 ml of contrast was then injected. CLO, AP, and lateral images of non-epidural spread were saved. The AP and CLO images were randomly mixed with images from historical controls with actual epidural spread.

**Results:**

A total of 24 false epidurograms in the AP and CLO views were mixed with an equal number of true epidurograms, resulting in 48 images each in the AP and the CLO views, respectively. Among the cohort of 10 experienced interventional pain physicians, the mean accuracy of correctly identifying epidural spread as epidural using the AP view alone was 51 % (SD 19 %). In addition, the accuracy of correctly identifying non-epidural spread as non-epidural using the AP view alone was 64 % (SD 15 %). Cohen's Kappa was 0.15, indicating minimal agreement between the interventionalists. In contrast, the mean accuracy of correctly identifying epidural spread as epidural using the CLO view alone was 99 % (SD 2 %). In addition, the accuracy of correctly identifying non-epidural spread as non-epidural using the CLO view alone was 96 % (SD 9 %). Excluding one outlier, the accuracy for the rest of the reviewers in determining non-epidural spread as non-epidural was 99 %. Cohens' Kappa was 0.95, indicating a high degree of agreement between the interventionalists.

**Conclusion:**

This study reveals that utilizing a standalone AP view without a CLO view was inadequate to distinguish epidural from non-epidural spread. Specifically, our study supports the continued use of CLO depth views to identify epidural contrast spread correctly.

## 1 Introduction

Epidural steroid injection (ESI) is a commonly performed interventional procedure for treating spinal pain [1]. The hallmark of this procedure is that the medication is correctly deposited in the epidural space. Fluoroscopy is helpful to ensure the medication is deposited at the correct level and laterality. In the absence of fluoroscopy, 53 % of the injections have been shown to be at the wrong level [2].

Whereas fluoroscopy is greatly useful in objectively determining the level and laterality, the essential technique while performing epidural access, whether fluoroscopy is used or not, relies on the loss of resistance at the point when the needle tip enters the epidural space. The rate of false loss of resistance (LOR) during epidural access is as high as 53 % [3].

It is clear, therefore, that just the LOR does not confirm that the needle tip has entered the epidural space; this is especially true in the anteroposterior (AP) view, where the operator cannot ascertain the depth of the needle tip and its relationship to the radiological landmarks such as the spinolaminar junction in the lateral view, or the ventral interlaminar line (VILL) in the contralateral oblique (CLO) view.

Contrast medium is injected to further ensure that the needle tip has entered the epidural space at the time of LOR, and the contrast pattern in the expected region further confirms its epidural location before the therapeutic agent is deposited.

Contrast patterns can be ubiquitous, and without knowledge of the spread depth, these may be inadequate to determine that the spread is epidural [[4], [5], [6]]. Given the chance of false LOR and the possibility of misidentifying the spread pattern, the AP view alone may not confirm epidural location. Thus, two views are recommended when performing an epidural steroid injection [7].

Nevertheless, proponents of the single AP view approach maintain that loss of resistance in the AP view and subsequent contrast spread patterns are sufficient to confirm epidural placement; routinely obtaining further views is unnecessary, adding time to the procedure and increasing the patient's radiation dose [8]. Indeed, the detractors of the single view approach are correct in pointing out that no clear evidence indicates that a lateral or CLO view confers any benefit [8].

This study analyzes the reliability of standalone AP and CLO views to confirm correct needle placement and put further speculation to rest. The authors hypothesize that the AP view may not reliably differentiate contrast spread inside the epidural space from superficial, non-epidural spread.

## 2 Methods

This single-center prospective study was conducted in an academic center's outpatient pain clinic in compliance with the relevant laws and institutional guidelines and approved by the Institutional Review Board (IRB Approval Date: 01/17/2023 and IRB Protocol number: 2022P000004/01). Twenty-five adult patients aged 18-years and older undergoing therapeutic lumbar interlaminar epidural steroid injections were included. Exclusion criteria encompassed pregnant patients and those with known allergies to iodinated contrast agents. Written informed consent was obtained from all study subjects before enrollment.

Patients were positioned prone for the procedure, and a 20-gauge Tuohy needle was aseptically advanced using the LOR technique under AP and CLO views. Before entering the epidural space, with the needle tip just posterior to the VILL in the CLO view, 0.5–1.0 ml of contrast agent, iohexol (omnipaque 300), was injected. Images of the contrast spread in AP, CLO, and lateral views were saved. The procedure was then completed by advancing the needle into the epidural space using LOR and confirming epidural spread, followed by administration of the therapeutic agent as clinically indicated. Other than an injection of contrast just before the VILL and obtaining the three images, the procedure adhered to standard protocols dictated by the clinical situation.

One subject was excluded as the contrast agent spilled into the epidural space at the time of extra epidural injection. A control group of 24 sets of images, each representing a loss of resistance in the epidural space in AP and CLO views with 0.5–1.0 ml of injected contrast dye, was randomly identified through a chart review of recent lumbar epidural steroid injections.

The images for the study and control groups were de-identified and presented to ten interventional proceduralists with a minimum of 5 years of clinical experience in pain medicine, who were blinded and who routinely performed or supervised interlaminar epidural injections in their practice. These clinicians were tasked with determining whether the images depicted epidural or non-epidural spread.

The survey questionnaire given to the interventionalists featured an equal number of images of epidural and non-epidural spread for each view, 96 images in total. They were tasked with determining whether each image represented epidural or non-epidural spread. Without any previous relevant data, this sample size of images was deemed sufficient to assess either view's validity in correctly distinguishing epidural from non-epidural spread. To minimize the subjective bias of a single interpretation, we included ten experienced interventionalists, each representing an objective and competent judge of the view.

We used Cohen's Kappa as the statistical measure to assess the level of agreement among the proceduralists beyond what would be expected by chance. It ranges from −1 to 1, where 1 indicates perfect agreement, 0 indicates agreement expected by chance, and negative values suggest less agreement than expected by chance. We report Cohen's Kappa and the overall accuracy for each proceduralist.

**Fig. 1:**
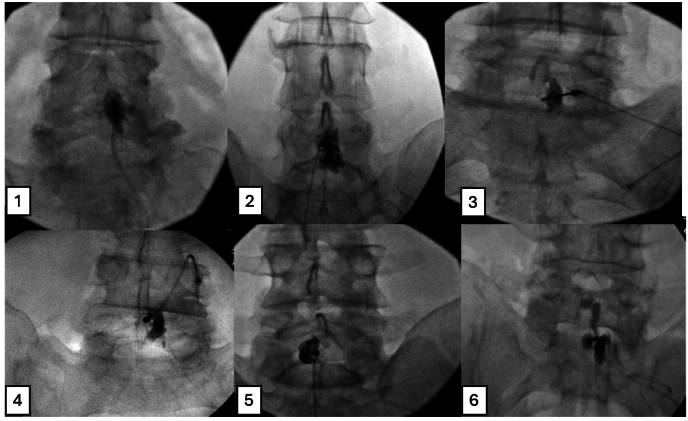
Presents fluoroscopic AP view images depicting the contrast medium spread pattern; see the key at the end of the article for clarification on whether the spread is within or outside the epidural space.

**Fig. 2:**
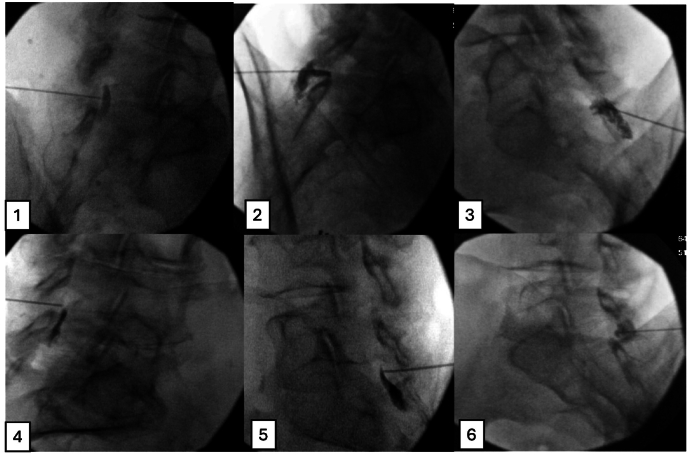
Presents fluoroscopic CLO view images depicting the contrast medium spread pattern; see the key at the end of the article for clarification on whether the spread is within or outside the epidural space.

Fig. 1 presents six fluoroscopic images in the AP view illustrating contrast medium spread patterns both within and outside the epidural space, while Fig. 2 shows six corresponding images in the CLO view. Refer to the answer key for details on the specific location of the contrast medium in each image.

## 3 Results

Among the cohort of 10 experienced interventionalists, the mean accuracy of correctly identifying epidural spread as epidural using AP view alone was 51 %, with a range of 21 %–79 % and an SD of 19 %. The mean accuracy of identifying non-epidural spread as non-epidural was 64 %, with a range of 46 %–100 % and an SD of 15 %. Cohen's Kappa was 0.15, indicating minimal agreement between the interventionalists (Table 1).

**Table 1:** Accuracy of each reviewer in correctly identifying epidural and non-epidural spread in AP (anteroposterior) view.

AP View	Epidural Identified as Epidural (24-Epidurograms)% Correct	Non-epidural Identified as Non-epidural (24-Epidurograms)% Correct	Cohen's Kappa
Reviewer 1	75 %	46 %	0.21
Reviewer 2	58 %	58 %	0.17
Reviewer 3	79 %	63 %	0.42
Reviewer 4	21 %	100 %	0.21
Reviewer 5	33 %	71 %	0.04
Reviewer 6	54 %	63 %	0.17
Reviewer 7	67 %	67 %	0.33
Reviewer 8	33 %	54 %	−0.13
Reviewer 9	46 %	54 %	0
Reviewer 10	46 %	67 %	0.13
Mean	51 %	64 %	0.15
SD	19 %	15 %	0.16

The mean accuracy of correctly identifying epidural spread as epidural spread using CLO view alone was 99 %, with a range of 96 %–100 % and an SD of 2 %. In addition, the accuracy of correctly identifying non-epidural spread as non-epidural was 96 %, with a range of 71 %–100 % and an SD of 9 %. Excluding one outlier (71 %), the accuracy for the rest of the reviewers in determining non-epidural as non-epidural was 99 %. Cohen's Kappa was 0.95, indicating high agreement between the interventionalists (Table 2).

**Table 2:** Accuracy of each reviewer in correctly identifying epidural and non-epidural spread in CLO (contralateral oblique) view.

CLO View	Epidural Identified as Epidural (24-Epidurograms)% Correct	Non-epidural Identified as Non-epidural (24-Epidurograms)% Correct	Cohen's Kappa
Reviewer 1	96 %	100 %	0.96
Reviewer 2	100 %	96 %	0.96
Reviewer 3	100 %	100 %	1
Reviewer 4	100 %	100 %	1
Reviewer 5	96 %	100 %	0.96
Reviewer 6	96 %	100 %	0.96
Reviewer 7	100 %	100 %	1
Reviewer 8	100 %	71 %	0.71
Reviewer 9	100 %	96 %	0.96
Reviewer 10	100 %	96 %	0.96
Mean	99 %	96 %	0.95
SD	2 %	9 %	0.09

**Fig. 3:**
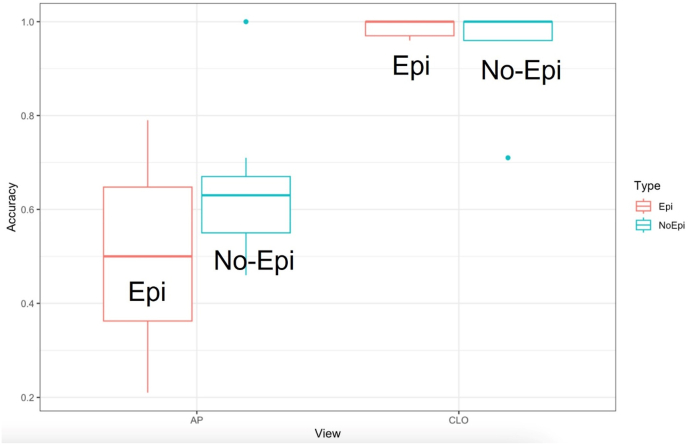
Box Plot shows the distribution of accuracy in each view. The median accuracy for CLO view is 100 % for both epidural and non-epidural images, and the interquartile range is very narrow.

Box Plot shows the distribution of accuracy in the AP and CLO views. The median accuracy for the CLO view is 100 % for both epidural and non-epidural images, with a narrow interquartile range (Fig. 3).

## 4 Discussion

The results of this study highlight the inadequacy of the AP view contrast spread pattern in distinguishing epidural spread from non-epidural spread. The accuracy of correctly identifying epidural spread as epidural was only 51 %, indicating a very high false negative rate. What was even more concerning was the fact that non-epidural spread was perceived as epidural 36 % of the time, indicating a very high false positive rate. Lastly, the very low level of agreement suggests that each reviewer made errors independently.

The results do not negate the utility of the AP view in performing a fluoroscopic epidural injection. Indeed, the AP view is the starting view and the gold standard for adhering to the correct side, but it cannot be used alone to confirm the epidural placement of the contrast agent.

The CLO view was remarkable in its accuracy in correctly determining epidural spread as epidural at 99 %; this is to be expected as the VILL is a very accurate radiological landmark; LOR cannot be anticipated before accessing the VILL [9,10]. Conversely, any contrast spread posterior to the VILL will be designated as non-epidural, as was seen in this study, with 96 % accuracy for identifying non-epidural spread as non-epidural, and excluding one outlier, the accuracy jumped to 99 %. Additionally, the reviewers had a high degree of agreement.

This study assessed the accuracy of these views in distinguishing epidural spread from non-epidural. In this small cohort, most of the epidural patterns were recognized as epidural in the contralateral oblique view since the only two options were epidural or non-epidural. Still, the authors also stress that contrast pattern recognition is an important skill, and even when the spread is at or anterior to the VILL, the possibility of intradural or subarachnoid spread should always be in the differential [5,6,[11], [12], [13], [14], [15]]. A review of preoperative 3D imaging can help make sense of contrast patterns when there is ambiguity.

Although lateral fluoroscopic images were collected in the study group, they were not compared to those in the control group, as lateral imaging was not routinely performed for depth determination. Instead, CLO depth imaging was routinely used for lumbar epidural access, and thus, lateral images were not available for the control group.

The study has significant limitations. Firstly, the images presented to the reviewers were not accompanied by any data. There are many components to the performance of an epidural steroid injection. The practitioner would get the characteristic feel during LOR; this, along with adequate insertion depth, would help determine whether epidural space has been accessed. Thus, the likelihood of a false negative or positive using a standalone AP view is likely much lower but also likely depends upon the practitioner's experience and expertise. Secondly, only 0.5 ml–1 ml of contrast was used; higher volumes could improve the ability to distinguish epidural from non-epidural spread. However, this supposition may be inaccurate and is unlikely to make such a dramatic difference that the standalone AP view could become reliable. Furthermore, the small volume in the CLO view was adequate for the distinguishment. Even though the sample size of this pilot study is small, the results clearly show that a larger sample size is not needed to determine the adequacy of the AP view. A more extensive study, however, can further establish the utility of the lumbar CLO view in distinguishing epidural from non-epidural spread.

This study concludes that lumbar AP contrast medium spread patterns without other data are unlikely to confirm epidural spread. Conversely, the CLO view appears highly reliable for distinguishing epidural from non-epidural contrast medium spread. Continued use of AP and CLO depth views is recommended for lumbar epidural access.

Refer to the answer key below for Fig. 1, Fig. 2:

Fig. 1 (AP View): 1-True, 2-True, 3-False, 4-False, 5-False, 6-True.

Fig. 2 (CLO View): 1-True, 2-False, 3-False, 4-True, 5-True, 6-False.

## Declaration of competing interest

The authors declare that they have no known competing financial interests or personal relationships that could have appeared to influence the work reported in this paper.
